# Donnan Dialysis for Recovering Ammonium from Fermentation Solutions Rich in Volatile Fatty Acids

**DOI:** 10.3390/membranes13030347

**Published:** 2023-03-17

**Authors:** Kayo Santana Barros, Mónica Carvalheira, Bruno Costa Marreiros, Maria Ascensão M. Reis, João Goulão Crespo, Valentín Pérez-Herranz, Svetlozar Velizarov

**Affiliations:** 1 LAQV/REQUIMTE, Department of Chemistry, NOVA School of Science and Technology, FCT NOVA, Universidade NOVA de Lisboa, 2829-516 Caparica, Portugal; 2 IEC Group, ISIRYM, Universitat Politècnica de València, Camí de Vera s/n, 46022, P.O. Box 22012, E-46071 València, Spain; 3 Associate Laboratory i4HB—Institute for Health and Bioeconomy, NOVA School of Science and Technology, Universidade NOVA de Lisboa, 2829-516 Caparica, Portugal; 4 UCIBIO—Applied Molecular Biosciences Unit, Department of Chemistry, NOVA School of Science and Technology, Universidade NOVA de Lisboa, 2829-516 Caparica, Portugal

**Keywords:** Donnan dialysis, cation-exchange membrane, ammonium sorption, ammonium limitation, biopolymer, protein-rich feedstocks, volatile fatty acids, organic acids recovery, biopolymers, PHA production

## Abstract

For the production of polyhydroxyalkanoates (PHA) using nitrogen-rich feedstocks (e.g., protein-rich resources), the typical strategy of restricting cell growth as a means to enhance overall PHA productivity by nitrogen limitation is not applicable. In this case, a possible alternative to remove the nitrogen excess (NH_4_^+^/NH_3_) is by applying membrane separation processes. In the present study, the use of Donnan dialysis to separate ammonium ions from volatile fatty acids present in the media for the production of PHA was evaluated. Synthetic and real feed solutions were used, applying NaCl and HCl receiver solutions separated by commercial cation-exchange membranes. For this specific purpose, Fumasep and Ralex membranes showed better performance than Ionsep. Sorption of ammonium ions occurred in the Ralex membrane, thus intensifying the ammonium extraction. The separation performances with NaCl and HCl as receiver solutions were similar, despite sorption occurring in the Ralex membrane more intensely in the presence of NaCl. Higher volumetric flow rates, NaCl receiver concentrations, and volume ratios of feed:receiver solutions enhanced the degree of ammonium recovery. The application of an external electric potential difference to the two-compartment system did not significantly enhance the rate of ammonium appearance in the receiver solution. The results obtained using a real ammonium-containing solution after fermentation of cheese whey showed that Donnan dialysis can be successfully applied for ammonium recovery from such solutions.

## 1. Introduction

Polyhydroxyalkanoates (PHA) are biobased and fully biodegradable polymers with excellent biocompatibility and are feasible substitutes of conventional plastics due to their similar physicochemical properties and a wide range of applications [1]. Therefore, the PHA industry is growing globally with a compound annual growth rate of 15.3%, and is estimated to reach a global market value of EUR 167 million by 2027 [2]. Nonetheless, the production cost of PHA is still estimated to be four times higher (EUR 4–5 /kg) than that of conventional plastics [1].

A promising PHA production strategy to reduce costs consists of using open mixed microbial cultures (MMC). This approach enables the use of inexpensive waste streams or by-products as feedstocks and does not require aseptic conditions, thus reducing the process energy demand [1] and facilitating the process protocol. The PHA production by MMC generally comprises three stages [1]: (1) acidogenic fermentation of the carbon feedstock into a volatile fatty acid (VFA)-rich stream, which are the precursors for PHA biosynthesis; (2) selection of an aerobic mixed culture enriched in PHA-accumulating organisms; and (3) PHA production using the culture selected in (2) fed with the fermented stream produced in (1).

PHAs are produced by specific microorganisms as intracellular carbon and energy reserves when cells are unable to grow at the same rate at which they take up carbon substrate. Therefore, restricting cell growth is the basis of PHA production, which can be achieved by means of nutrient limitation (e.g., lack of nitrogen or phosphorous). The PHA production stage is preferably carried out in the absence of nutrients to reach its maximum PHA capacity, as their presence might channel a fraction of the available carbon towards cell growth. Nonetheless, the culture selection is carried out in the presence of nutrients in order to sustain growth of the enriched culture with PHA-accumulating organisms [1]. Therefore, the culture selection stage is considered the core of the process, as the obtained PHA-accumulating MMC determines the global volumetric PHA productivity, and subsequently, the effectiveness of the PHA production process. 

The enrichment of a MMC with PHA-accumulating organisms is essentially based on imposing an internal growth limitation, achieved by applying the so-called feast and famine (F/f) regime. The F/f regime consists in subjecting the culture to cycles of alternated periods of carbon excess (feast phase) and carbon absence (famine phase). By doing so, during the famine phase, PHA accumulating microorganisms use external nitrogen and stored PHA for their growth and maintenance. Additionally, prolonged periods of carbon limitation cause limitations of the internal growth factors, making the cells store PHA instead of immediate growth when exogenous carbon is available in the subsequent F/f cycle [1].

When using nutrient-poor feedstocks (e.g., fruit waste [3], candy bar factory wastewater [4], paper mill wastewater [5]) for PHA production, the culture selection can further be improved by controlling the nitrogen availability in the F/f cycle. Previous studies [6,7] demonstrated that subjecting the culture to an uncoupled C/N feeding (i.e., nitrogen is introduced exclusively during the famine period) benefits culture enrichment and thereby the potential of PHA accumulation. However, when using feedstocks that are not nutrient-poor (e.g., protein-rich feedstocks [8,9]), the above-mentioned strategy cannot be used, nor can the PHA production stage can be carried out in the absence of nutrients. Therefore, to allow the uncoupled C/N feeding and, consequently, improve the culture enrichment and PHA production, dedicated strategies to remove or decrease the nitrogen content in the feedstock should be implemented. A promising approach for this purpose is the use of membrane separation techniques, such as electrodialysis [10], forward osmosis [11], gas-permeable membrane contactors [12], and membrane extraction [13]. Another membrane process that can be used for this purpose is Donnan dialysis, which is an environment-friendly process that allows for the separation and recovery of target ionic species. Among the possible membrane techniques, Donnan dialysis stands out due to the simplicity of its layout, operation, and maintenance. In addition, as it does not operate under application of pressure or an externally applied electric potential difference, the occurrence of the intense fouling/scaling phenomena is mitigated [14]. Despite the advantages of using Donnan dialysis, to the best of our knowledge, there are no reported studies in the open literature on its use to separate ammonium and VFAs from fermentation solutions.

Donnan dialysis (DD) is a separation process using an ion-exchange membrane separating a compartment containing the solution to be treated (feed) from a compartment with the solution that receives the target ions (receiver). Ion transport is governed by an electric potential difference established due to the concentration gradients of ions to be transported through the membrane (counter-ions) present at each of its sides. In general, the receiver solution is composed of a salt (e.g., NaCl or KCl), a strong acid (e.g., HCl or H_2_SO_4_), or a strong base (e.g., NaOH or KOH) at a concentration considerably higher than the concentration of the target counter-ions to be extracted from the feed solution. In this sense, counter-ions from the receiver solution, referred to as “driving” counter-ions, are transported to the feed solution forcing counter-ions from the feed to be transported in the opposite direction until the Donnan equilibrium is reached. Although DD offers several advantages, the process also presents some limitations, such as relatively long operating times and the requirement of large membrane areas [15,16]. Thus, dedicated optimization studies need to be conducted in order to make its practical application feasible.

The use of DD is often evaluated for treating water/wastewater containing ionic contaminants and/or resources, such as copper, fluoride, nitrate, arsenate, aluminum, gold, lithium, and phosphate [17,18]. In the last few years, several authors have evaluated the use of DD to separate ammonium ions from solutions that simulated water/wastewater and obtained promising results. However, some of these studies have been conducted with synthetic feed solutions with only NH_4_^+^-containing compounds, which do not correspond to the compositions used in a real process [18,19].

The aim of this study is to assess the feasibility of using DD for the treatment of VFA-rich streams with an undesirably high ammonium ion content for the production of PHA by MMC. The primary objective is to develop a sustainable process that allows for both the recovery of a VFA-rich stream with low ammonium levels and an ammonium-rich solution that can be utilized at various stages of the PHA production process. Specifically, the VFA-rich stream can be utilized as a carbon source during culture selection and PHA production, while the ammonium-rich solution can be used as ammonium supplementation during the famine phase of the F/f cycle to stimulate the growth of PHA-accumulating organisms.

The study was conducted first using a synthetic feed solution containing ammonium, phosphate, VFAs (acetic, propionic, butyric, and valeric acids), and trace elements (copper, iron, molybdenum, and nickel). The effects of the type of cation-exchange membrane, type and concentration of counter-ions in the receiver solution, solution flow rate, volume ratio of feed:receiver solutions, and application of an external electric potential were evaluated. Before evaluating the effect of an external electric potential, current–voltage curves of the membrane/solution systems were obtained. Then, experiments using a real feed solution obtained from acidogenic fermentation of cheese whey were carried out applying distinct conditions, which were determined from the experiments conducted with synthetic solutions.

## 2. Materials and Methods

### 2.1. Donnan Dialysis Apparatus 

The DD experiments were conducted using a module with two identical rectangular channels, separated by a commercial cation-exchange membrane under study. The membrane had an active area of 41.6 cm^2^ and was horizontally positioned. One of the module channels was connected to an external loop where the solution to be treated, initially rich in VFAs and ammonium ions, was recirculated. This compartment is labeled as the feed, while the compartment connected to the other module channel, containing the solution that received ammonium, is labeled as the receiver. The two solutions were recirculated between the module and the respective reservoirs independently, by means of peristaltic pumps. Both reservoirs were stirred by magnetic bars. The volume of the feed solution was 1 L, while for the receiver solution it was 1 L or 2 L. Aliquots of 8 mL were withdrawn from both compartments throughout the experiments to determine the concentrations of ammonium, phosphate, organic acids, and trace metals, in addition to measuring the pH of the solutions. The following operating parameters were evaluated: membrane type (Ralex CMH-PES, IONSEP-HC-C, and Fumasep FKS-PET 130), receiver solution type (NaCl and HCl), solution flow rate (110–440 mL/min), concentration of receiver solution (0.125 M, 0.25 M, and 0.5 M), volume ratio of feed:receiver solutions (V_F_:V_R_) (1:1 and 1:2), and type of feed solution (synthetic and real solution from a fermentation process). The experiments were conducted in duplicate and at air-conditioned room temperature (~20 °C).

### 2.2. Two-Compartment Apparatus Operated under External Electric Potential Application

The experiments operated under external electric potential application were carried out in a two-compartment plexiglass cell with a cation-exchange membrane separating the two compartments with a volume of 175 mL each, containing, respectively, the feed and receiver solutions. This two-compartment system operating under an external electric potential application is commonly referred to as membrane electrolysis since reduction and oxidation reactions, such as hydrogen evolution and water electrolysis, occur at the electrodes. In the present paper, the term membrane electrolysis was considered only for the cases where the values of applied electric potential were high enough for the occurrence of electrochemical reactions at the electrodes. The membrane used was Ralex CMH-PES, which had an active area of 11.2 cm^2^ and was vertically positioned. Each compartment was connected to external reservoirs containing the feed and receiver solutions, which were stirred with magnetic bars and circulated through the cell by independent peristaltic pumps. The working (cathode) and counter (anode) electrodes were Ag rods having diameter of 5 mm, purity of 99.95%, and an active area of 9 cm^2^ (GoodFellow Company, Huntingdon, UK). The electrodes were positioned in the center of each compartment and connected to a potentiostat/galvanostat (Autolab PGSTAT204, Utrecht, The Netherlands) that supplied the electric potential. The electrode present in the receiver solution compartment was the cathode, while the electrode in the feed solution compartment was the anode. The experiments were conducted without external electric potential application and also at −0.1 V, −0.6 V, and −1.0 V, in duplicate and at a room temperature (~25 °C). Table 1 presents the experimental conditions of all DD tests conducted in the present study.

### 2.3. Current–Voltage Curves of the Membrane/Electrolyte Systems

Current–voltage curves of the membrane/electrolyte systems were constructed by linear sweep voltammetry at a scan rate of 0.002 V/s using a system similar to that described in Section 2.2. The cation-exchange membrane used was Ralex, which was chosen based on the results of the tests conducted without external electric potential application, and the solutions were NaCl 0.25 M (receiver) and the synthetic feed solution. Two Ag rods were used as working and counter-electrodes, which were positioned at the center of each compartment. Platinum wires placed very close to the cations depleted and enriched interfaces of the membrane were used as reference electrodes to measure the membrane potential drop under electric current application. The curves were constructed using two configurations concerning the solutions present in each of the compartments. First, a curve was obtained with the synthetic feed solution present in both compartments, that is, (1) synthetic feed solution—membrane—synthetic feed solution. This curve was obtained to eliminate the effect of the concentration difference between the compartments for evaluating the transfer of NH_4_^+^ ions governed only by the electric potential difference. Then, a curve was obtained using the DD configuration with different solutions in the compartments, that is, (2) receiver solution (0.25 M NaCl)—membrane—synthetic feed solution. The experiments were conducted in duplicate and at a room temperature (~25 °C).

From the curves obtained, which presented the three distinct regions, the limiting current density (*i_lim_*) of the membrane/electrolyte system, ohmic resistance (*R*_Ω_), and plateau length were determined, as described in detail by Barros et al. [20]. The *i_lim_* was obtained by the intersection of the tangential line of the first and second regions of the curve (quasi-ohmic and plateau regions, respectively). The *R*_Ω_ was determined from the inverse of the slope of the tangential line of the quasi-ohmic region. The plateau length was determined by subtracting the final and initial potential values of the second region of the curve (plateau region).

### 2.4. Ion-Exchange Membranes

Three cation-exchange membranes were evaluated in the experiments: Ralex CMH-PES (MemBrain s.r.o, Stráž pod Ralskem, Czech Republic), IONSEP-HC-C (Hangzhou Iontech Environmental Technology Co., Ltd., Hangzhou, China), and Fumasep FKS-PET-130 (Fumatech BWT GmbH, Bietigheim-Bissinge, Germany). The main characteristics of the membranes are shown in Table 2. Although the main characteristics of Ralex CMH-PES and IONSEP-HC-C membranes are similar, they were tested in this work because Welter [21] showed that the difference in the amount and area occupied by the ion-exchange particles of the functional groups of Ralex are greater than that of Ionsep, which can affect the transfer of ions through the membranes. Before the experiments, the membranes were converted into Na^+^-form in a 0.5 M NaCl solution (renewed several times, as recommended by the manufacturers) for 72 h. Afterwards, they were rinsed with distilled water.

### 2.5. Working Solutions

The study was conducted using synthetic and real feed solutions. First, synthetic feed solutions were used to evaluate the influence of the main operating parameters on the separation of ammonium ions. Then, the DD system was operated with a real feed solution from a fermentation process to evaluate the applicability of a real process when applying different conditions. In all experiments, a NaCl or a HCl containing aqueous solution was used as a receiver solution.

#### 2.5.1. Synthetic Feed Solution

The synthetic feed solution was freshly prepared with distilled water, NH_4_Cl (Biochem Chemopharma, Cosne-Cours-sur-Loire, France), KH_2_PO_4_ (Chem Lab, Zedelgem, Belgium), and the following VFAs: acetic acid (Fisher Scientific, Loughborough, UK), propionic acid, butyric acid, and valeric acid (Acros Organics, Geel, Belgium), which serve as the PHA precursors. In addition, a trace elements solution containing CuCl_2_·2H_2_O, Na_2_MoO_4_·2H_2_O, NiCl_2_·6H_2_O, and FeCl_2_·4H_2_O was added in order to mimic a real fermented stream used in the PHA production. The solution pH was adjusted to 4.5 using NaOH (Fisher Scientific, Loughborough, UK). The composition of the synthetic feed solution is shown in Table 3. The Cmol:Nmol ratio was set to 1:1.

#### 2.5.2. Real Feed Solution

The real feed solution was obtained from the acidogenic fermentation of cheese whey using a continuous stirred-tank reactor and operated under an organic loading rate of 20 g of cheese whey/(L·day), pH of 4.5, and at 30 °C. The composition of the real feed solution is shown in Table 4. Note that the Cmol:Nmol ratio was 8.5. The high concentration of Na^+^ and Cl^−^ ions was due to the use of NaOH and HCl, respectively, for adjusting the pH of the fermentation solution.

#### 2.5.3. Receiver Solution

NaCl (0.125 M, 0.25 M, and 0.5 M) and HCl (0.25 M) were tested as receiver solutions, which were prepared with NaCl (Panreac, Barcelona, Spain) or HCl (Fisher Scientific, Loughborough, UK) and distilled water. For the NaCl receiver solution, the Na^+^ ions presented molar concentrations of 5 (0.125 M), 10 (0.25 M), and 20 (0.5 M) times greater than that of the NH_4_^+^ ions (0.025 M) in the feed, respectively, whereas for the HCl solution, the molar concentration of H^+^ ions (0.25 M) was 10 times greater than that of NH_4_^+^ ions in the feed.

### 2.6. Analytical Methods

Total suspended solids (TSS) and volatile suspended solids (VSS) were quantified using the standard methods [28]. The concentration of ammonium and phosphate ions was determined through a colorimetric method with a segmented flow analyzer (Skalar San++, Skalar Analytical, Breda, The Netherlands). The concentration of organic acids was determined by high-performance liquid chromatography (HPLC) in a VWR Hitachi Chromaster chromatographer (Hitachi, Tokyo, Japan) equipped with a Pump 5160, an auto sampler 5260, a Column Oven 5310, a Diode Array Detector 5430, a RI Detector 5450, a Biorad 125-0129 30 × 4.6 mm pre-column, and an Aminex HPX-87H 300 × 7.8 mm column (0.01 M H_2_SO_4_ eluent, flow rate 0.6 mL/min and column temperature 60 °C). The concentrations of trace and other cationic species, such as sodium and potassium ions, were determined by means of Inductively Coupled Plasma-Atomic Emission Spectrometer—ICP-AES (Horiba Jobin-Yvon Ultima, Longjumeau, France), equipped with a 40.68 MHz RF generator, Czerny-Turner monochromator with 1.00 m (sequential), autosampler ASS00 and CMA (Concomitant Metals Analyzer). The concentrations of trace and other anionic species, such as chloride and fluoride, were determined by an Ion Chromatography System ICS3000 (DIONEX, Sunnyvale, CA, USA). The system was composed of an IR-detector, a pre-column (Ionpac AG9-HC), and a column (Ionpac AS9-HC) at 25 °C. The eluent was NaCO_3_ (9 mM) with a flow rate of 1 mL/min using a standard concentration range of 10–100 mg/L. The pH of the solutions was measured using a pH meter Sension+ pH3 (Hach, Loveland, CO, USA). The structure of the membranes’ surface was evaluated by means of Attenuated Total Reflection Fourier Transform Infrared Spectroscopy, ATR—FTIR (Spectrum Two FT-IR Spectrometer, PerkinElmer, Walthan, MA, USA).

### 2.7. Calculations

The percent extraction (PE%) of ammonium ions extracted from the feed solution was calculated using Equation (1), where $C_{F_{0}}$ and $C_{F_{f}}$ are the ammonium concentration in the feed solution in the initial and final states, respectively.

(1) $$PE\left(\%\right)=\left(1-\frac{C_{F_{f}}}{C_{F_{0}}}\right)\times 100$$

The flux (*J*) of ammonium ions reaching the receiver compartment was calculated using Equation (2), where $C_{R_{0}}$ and $C_{R_{i}}$ are ammonium concentration in the receiver solution in the initial state and at time *i,* respectively, *V* is the receiver solution volume, $A_{m}$ is the membrane area and *t* is the running time. As ion flow varies as a function of time due to the driving force reduction, the flux values shown herein refer to the arithmetic mean of the first two values obtained for each membrane (at 1.9 h and 4.7 h for Ralex, 2.1 h and 5.5 h for Ionsep, 2.5 h and 5.5 h for Fumasep).

(2) $$J=\frac{\left(C_{R_{i}}-C_{R_{0}}\right)V}{A_{m}t}$$

The theoretical mass balance of ammonium ions in each experiment was calculated using the volume and concentration of the withdrawn aliquots and the theoretical volume of the solutions in both compartments (considering the withdrawn aliquots) at different operating times (*i*), as shown in Equations (S1)–(S3) (Supplementary Materials). As the effect of water transfer through the membranes was not considered in the theoretical mass balance, the real/final mass balance (MB) was also calculated considering the volume and concentration of the withdrawn aliquots and the real/final volumes of the solutions in both compartments at the end of the experiments, as shown in Equations (S2), (S4) and (S5). 

### 2.8. Evaluation of Ammonium Sorption in the Membranes

The occurrence of ammonium sorption in the Fumasep, Ralex, and Ionsep membranes was evaluated. The tests were conducted with HCl and NaCl solutions. First, membrane samples with equal areas were weighted. Then, they were immersed in a beaker with 100 mL of the synthetic feed solution for 48 h. The membranes were rinsed with distilled water for 8 h to remove any trace of the feed solution from their surfaces. Lastly, the membranes were immersed in a beaker with 100 mL of a 0.25 M NaCl or 0.25 M HCl solution for 48 h. After this procedure, the feed and NaCl or HCl solutions were analyzed for determining the ammonium concentration before and after each stage. The tests were performed in duplicate for each membrane and the results shown refer to the arithmetic means obtained. The errors were below 3%.

## 3. Results and Discussion

### 3.1. Evaluation of the Membrane Type

#### 3.1.1. Evaluation of Ammonium Transfer

The performance of Ralex CMH-PES, IONSEP-HC-C, and Fumasep FKS-PET-130 membranes in Donnan dialysis to separate ammonium from organic acids and phosphate was evaluated using a receiver solution composed of NaCl at 0.25 M. The tests were conducted for 80 h. 

**Figure 1:**
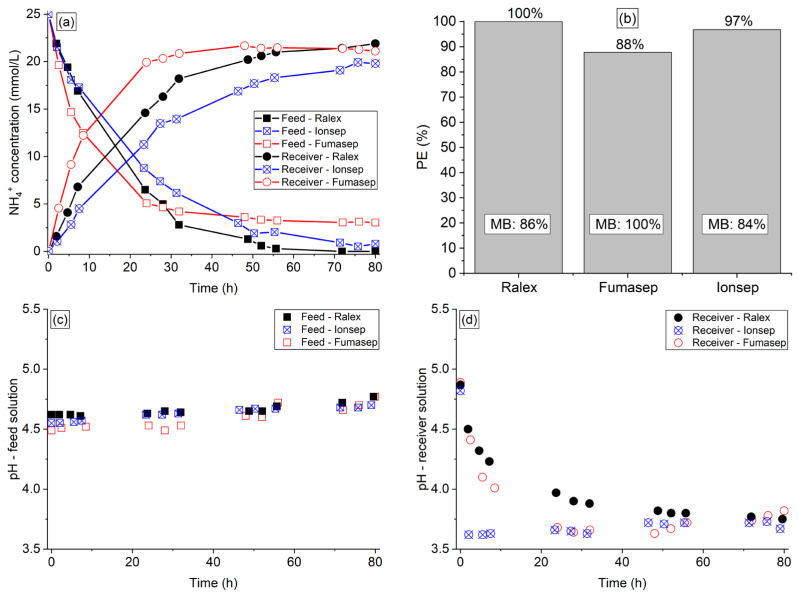
(**a**) NH4^+^ concentration profile, (**b**) percent extraction, mass balance, (**c**) pH profile of the synthetic feed, and (**d**) receiver solutions of the experiments conducted with Ralex, Ionsep, and Fumasep membranes.

The concentrations of organic acids and phosphate ions present in the feed solution were not changed throughout the experiments; therefore, they were not transported to the receiver solution. This behavior was expected since the organic acids are neutral and negatively charged species at the initial feed pH of 4.5 (pKa of 4.76, 4.87, 4.84, and 4.82 for acetic, propionic, butyric, and valeric acids, respectively), whereas phosphate ions have a negative charge [29]. The results of ammonium concentration in both compartments are shown in Figure 1a, whereas the results of NH_4_^+^ percent extraction and real/final mass balance (MB) are shown in Figure 1b. The pH vs. time profiles of both solutions are shown in Figure 1c,d.

The membranes showed distinct ammonium separation performances. Considering the NH_4_^+^ concentration profiles in the receiver solution (Figure 1a), the ammonium transfer rate followed the order of Fumasep > Ralex > Ionsep (initial ammonium flux of $4.1\times 10^{-5}mol/(cm^{2}\cdot h),2.0\times 10^{-5}mol/(cm^{2}\cdot h),1.2\times 10^{-5}mol/(cm^{2}\cdot h)$, respectively). Note that the ammonium removal from the feed was considerably faster with Fumasep than Ralex and Ionsep. This is also noticeable by evaluating the intersection point of ammonium concentrations in the feed and receiver solutions, besides the time taken to reach the Donnan equilibrium conditions. This difference between ion transfer through the membranes can be explained by their characteristics shown in Table 2. It is well known that high ion-exchange capacity (IEC), high water content, and low thickness values enhance ion transport through ion-exchange membranes [30]. Although the IEC and water content of Ralex and Ionsep membranes are considerably greater than that of Fumasep, the transport of counter-ions through the latter was faster due to its considerably lower thickness. Thus, the results suggest that the thickness and homogeneity had a greater influence on ammonium transfer through the studied membranes than their IEC and water content.

With regard to the ammonium concentration profile in the feed compartment, the curves in Figure 1a showed an unexpected behavior. Note that during the first hours of experiment (approximately 25 h), the ammonium transfer rate followed the same behavior of the receiver compartment, as expected: Fumasep > Ralex > Ionsep. However, for times longer than 25 h for Ralex and 45 h for Ionsep membranes, the ammonium concentration became lower than with Fumasep, reaching ammonium percent extractions of 100% (Ralex), 97% (Ionsep), and 88% (Fumasep) after 80 h of operation—Figure 1b. As the decrease in concentration of the feed solution with Ralex and Ionsep occurred in a different proportion of the receiver solution, this indicates that when both membranes were used, part of NH_4_^+^ ions left the feed compartment but did not reach the receiver solution, which may have occurred due to their sorption in the membranes. In order to confirm this assumption, the theoretical (Table S1 of Supplementary Materials) and real mass balance (Figure 1b) were calculated for each experiment. Note that the mass balance reached 100% only for the system with the Fumasep membrane, confirming that sorption has occurred in the Ralex and Ionsep membranes (ammonium mass balance of 86% and 84%, respectively). Although Ralex and Ionsep membranes presented a lower ion transfer rate, the sorption phenomenon may be exploited in the process since the ammonium extraction was intensified. The different behavior related to ammonium sorption in the membranes can be explained by their thickness (Table 2) and structure, as will be discussed in Section 3.1.2. It is worth mentioning that sorption depends not only on the membrane type, but also on the counter-ions type and receiver solution concentration, but most importantly on the operation mode [31,32]. Sorption is a phenomenon of practical interest mostly for batch DD process operations since in a continuous operation, the membranes reach a saturation level, thus making sorption effects relevant only in the process initial stage.

As ion-exchange membranes are not perfectly permselective, they also allow the transport of water by osmosis as a result of osmotic pressure differences between the diluted and concentrated solutions [33]. Therefore, the volumes of the withdrawn aliquots and the remaining solutions in the reservoirs were analyzed. It was verified that this phenomenon occurred more intensely with the Fumasep membrane, which showed a deviation of +11.2% in relation to the expected volume in the receiver solution, whereas for Ralex and Ionsep the deviation was lower than 3%. This can be explained by the difference in their water content values (Table 2), since the intensity of water transfer through membranes depends strongly on this property [34].

According to Figure 1c, the pH of the feed solution increased throughout the experiment, while for the receiver solution (Figure 1d) it decreased. The pH reduction of the receiver solution with Fumasep was considerably more intense in the first hours when compared to the other membranes because, as shown in Figure 1a, the ion transport through this membrane was more intense in the beginning of the experiment. The pH changes of both solutions throughout the experiment can be explained by the acidity of the NH_4_^+^ ions that left the feed solution and reached the receiver one. Note in Figure 1d that the pH of the receiver solution became practically constant at a given time, which coincided with the ammonium concentration profile shown in Figure 1a. By evaluating the initial and final pH values of both solutions, one can note that the pH variation occurred more strongly in the receiver compartment. This occurred because the receiver solution was composed of a neutral salt, whereas the feed solution was a complex solution that may have buffering properties. Therefore, the NaCl solution was more affected by the presence of the transferred NH_4_^+^ ions. 

#### 3.1.2. Membrane Characterization: Evaluation of Ammonium Sorption and Their Structure by ATR-FTIR

As described in Section 2.8, the occurrence of ammonium sorption in the membranes was evaluated, and the results are shown in Table S2. The table presents the ammonium concentration in the feed and NaCl/HCl solutions before and after each procedure. The mass balance of ammonium ions was also performed considering its concentration in the initial and final solutions. Note that Fumasep did not sorb ammonium in both tests, whereas Ralex and Ionsep membranes sorbed the same amount of ammonium ions per mass of dry membrane (~1.2 mmol/g). After the NaCl regeneration step, most of the ions sorbed in Ralex were desorbed since the percentage of final mass balance was 96.1%. On the other hand, the mass balance for Ionsep remained relatively far from 100%, even after the regeneration step with NaCl (92%), which could impair the ion separation because of reduction in the ion-exchange capacity of the membrane functional groups in long-term operations. The regeneration procedure with HCl showed results slightly better than those with NaCl only for the Ionsep membrane. Thus, both solutions can be used to regenerate Ralex and Fumasep membranes after each batch of DD to be converted into their initial Na^+^ form, whereas HCl is more appropriate to regenerate the Ionsep membrane. It is suggested that it would be more practical if the solution used to regenerate the membrane from a previous batch is the same that will be used as the receiver solution in the next batch. In this sense, the NaCl or HCl solution would be used as regenerating and receiver solutions, allowing the recovery of sorbed ammonium ions in the receiver solution.

A structural characterization of the Fumasep, Ralex, and Ionsep membranes was conducted by ATR-FTIR to evaluate the influence of their polymer and functional groups on the ion transfer and occurrence of ammonium sorption in each of the membranes. The IR spectra of the membranes are shown in Figure S1, and a detailed description of the peaks is shown in Section S1 of Supplementary Materials. It was verified that the intensity of the peaks between 1173–1008 cm^−1^, which are related to the -SO_3_^−^ functional groups, is considerably higher for the Ralex membrane when compared to Ionsep. This may have influenced the faster ion transfer through the former than the latter (Figure 1a) due to the greater attraction of cations by the functional groups for Ralex. It was also verified that the bands related to the functional groups of Fumasep were split into several smaller peaks between 1219–1022 cm^−1^, which can be explained by its homogeneity and the nonexistence of agglomerates of ion-exchange particles typical of heterogeneous membranes [30]. This may have influenced the lower sorption degree of NH_4_^+^ ions in Fumasep, in addition to its lower thickness.

As Ionsep presented the lowest ion transfer rate (Figure 1a) and offered greater resistance to desorb NH_4_^+^ ions, its use in the DD process evaluated herein must be avoided. Concerning Ralex and Fumasep membranes, the former has a considerably lower market price than the latter, which can make its use more advantageous. In addition, the sorption phenomenon that occurred in Ralex increased the extraction of NH_4_^+^ from the feed solution, which could be exploited in the separation process. Note in Figure 1a that although the initial rate of ion transfer was higher with Fumasep, the NH_4_^+^ concentration in the feed after 30 h of operation was lower with Ralex. On the other hand, if Ralex is used, NH_4_^+^ ions need to be desorbed/recovered from the membrane after each batch to avoid lowering the ion-exchange capacity of the membrane, which would harm the next batches. Based on the above-mentioned discussions, the experiments of the next sections will be conducted with the Ralex membrane. As the intensity of ammonium transfer through this membrane was initially lower than that through Fumasep, operational conditions capable of overcoming this limitation will be sought to make Ralex a viable membrane for the process evaluated in this study.

### 3.2. Evaluation of the Receiver Solution Type: HCl and NaCl

**Figure 2:**
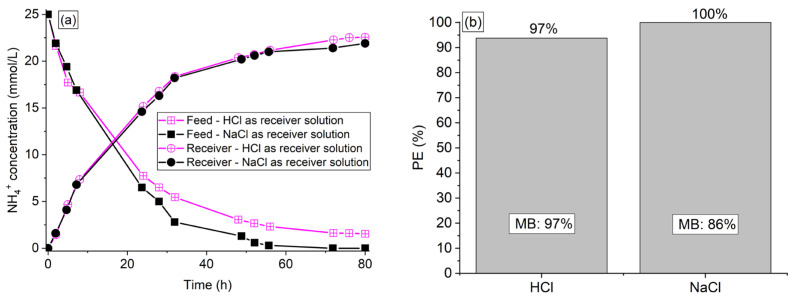

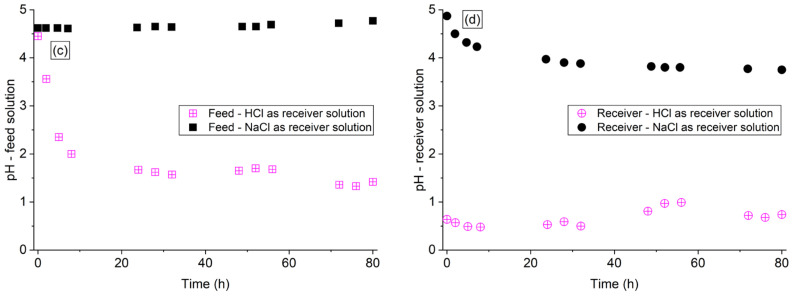
(**a**) NH4^+^ concentration profile, (**b**) percent extraction, mass balance, (**c**) pH profile of the synthetic feed, and (**d**) receiver solutions of the experiments conducted with HCl 0.25 M and NaCl 0.25 M as the receiver solution.

The influence of the receiver solution type was evaluated using HCl and NaCl solutions at 0.25 M with Ralex membrane for 80 h. The organic acids and phosphate ions remained in the feed solution. The results of ammonium concentration in both compartments are shown in Figure 2a, whereas the results of percent extraction and final mass balance are shown in Figure 2b. The pH vs. time profiles of the solutions are shown in Figure 2c,d. 

According to Figure 2a, the amount of NH_4_^+^ that crossed the membrane and reached the receiver solution was very similar with NaCl and HCl solutions during the entire operation. On the other hand, the concentration profile of the feed solution showed a different behavior especially at longer times (after approximately 20 h), since the NH_4_^+^ concentration in the feed solution was lower when using NaCl instead of HCl. As discussed in the evaluation of membrane type (Section 3.1.1), this indicates that part of the NH_4_^+^ ions were sorbed in the Ralex membrane when NaCl was used, which was confirmed by the theoretical (Table S3) and real mass balance (Figure 2b). This difference between the NaCl and HCl solutions was only verified at longer times because, at the beginning of the operation, the transport driving force of ion transfer was much higher since the solutions in each of the compartments presented a greater concentration difference. The greater ability of H^+^ ions from HCl to desorb NH_4_^+^ ions agrees with the subtle difference between NaCl and HCl solutions verified in the sorption phenomenon evaluation (Section 3.1.2). This may be explained by the smaller hydrated radius of H^+^ than Na^+^ ions (2.82 Å and 3.58 Å, respectively [35]), and the higher diffusivity and membrane selectivity for H^+^ than for Na^+^ [36]. Although Na^+^ and H^+^ ions show different hydration numbers, which could affect the water transfer across the Ralex membrane [34,37], this phenomenon was negligible with both receiver solutions (volume deviations below 3% in relation to the expected values).

As shown in Figure 2c,d, the pH profile for the NaCl solution was the same as that presented in Section 3.1. In this case, the increase in pH of the feed solution and a pH decrease for the receiver solution occurred due to the influence of NH_4_^+^ acidity, as already discussed. When HCl was used, the effect of this phenomenon became negligible since the migration of H^+^ from the receiver to the feed solution was intense. In this sense, the pH of the feed decreased significantly, while the pH of the receiver increased slightly.

Due to the similar performance obtained with HCl and NaCl solutions, especially at short operating times, in addition to the lower cost and corrosivity of NaCl, the experiments reported in the next sections have been carried out with NaCl solution at 0.25 M in the receiver compartment. As it is known, one of the main disadvantages of DD is its relatively long operating time since ion transfer occurs by counter-diffusion [15,18]. In order to overcome this limitation, the experiments presented in the next sections were carried out for 30 h, and methods attempting to enhance the counter-ions transfer were evaluated. As shown in Figure 2a, the type of receiver solution practically did not affect the intensity of ammonium transfer at short times, indicating that NaCl can be used without hindering the ion separation for 30 h. As it was suggested in Section 3.1.2, the membrane was regenerated with a NaCl solution at 0.25 M after each batch of DD to be converted into their initial Na^+^ form.

### 3.3. Effect of Flow Rate

The influence of flow rate of the solutions in each compartment on the NH_4_^+^ transfer through the Ralex membrane was evaluated for 30 h at flow rates in the range of 110 to 440 mL/min. For the module used (with a hydraulic channel diameter of 0.5 cm), this corresponded to superficial linear flow velocities of 4.1 to 16.4 cm/s and Reynolds number (Re) values of 205 to 820. This range was selected based on a previous study on nitrate removal by Donnan dialysis showing that concentration polarization effects within the liquid diffusion boundary layer at the membrane surface are strongly (>90%) minimized at Re of 205 and almost completely (>99%) avoided for Re > 800 [38]. Since the diffusion coefficient of NH_4_^+^ (1.96 × 10^−5^ cm^2^/s) in water at 25 °C is very similar to that of NO_3_^−^ (1.90 × 10^−5^ cm^2^/s) [39], it was considered reasonable to expect a similar contribution of the presence of a stagnant liquid diffusion boundary layer at the membrane/feed solution interface on the transport rates of these ions across it in the Donnan dialysis module used. 

**Figure 3:**
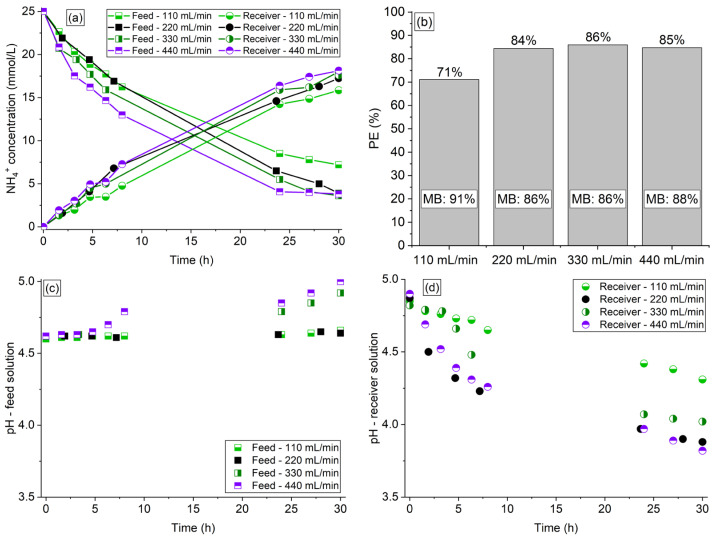
(**a**) NH4^+^ concentration profile, (**b**) percent extraction, mass balance, (**c**) pH profile of the synthetic feed, and (**d**) receiver solutions of the experiments conducted at flow rates of 110, 220, 330, and 440 mL/min.

The organic acids and phosphate ions remained in the feed solution. The results of ammonium concentration in both compartments are shown in Figure 3a, whereas the results of NH_4_^+^ percent extraction and final mass balance are shown in Figure 3b. The pH vs. time profiles of the solutions are shown in Figure 3c,d.

The increase in flow rate between 110–220 mL/min expectedly intensified the ammonium transfer (Figure 3a) due to the decrease of the thickness of liquid diffusion boundary layers at the membrane interfaces, especially the depleted one (membrane—feed solution) [40]. The curve for 220 mL/min showed a considerable difference in relation to the curves for 330 and 440 mL/min only during the first operating hours, since at longer times, the concentrations were practically identical, reaching the same percent extraction (~85%) after 30 h. The curves obtained at flow rates of 330 and 440 mL/min were very close, especially for the receiver solution, indicating that from 330 mL/min, a further increase in flow rate did not intensify the ammonium transfer. These results differ from those obtained by Chen et al. [18], who found that the effect of flow rate was negligible in the transfer of NH_4_^+^ ions under all conditions tested. This may have occurred because the flow rates tested by those authors were considerably higher so that the ammonium transfer was not altered, as occurred in the present work for the 330 mL/min and 440 mL/min curves. As shown in Figure 3b and Table S4, the increase in flow rate did not affect considerably the mass balance of NH_4_^+^ ions especially within the range of 220–440 mL/min. The intensity of water transfer through the membrane was not influenced by the flow rate, showing volume deviations below 3%. 

Note in Figure 3c,d that the increase in flow rate caused a pH behavior similar to that presented in the previous sections: an pH increase in the feed and a pH decrease in the receiver solution. Here, the intensity of the pH increase/decrease did not show a direct relationship with the intensity of NH_4_^+^ transfer, especially for the receiver solution (compare Figure 3a with Figure 3c,d).

As the NH_4_^+^ percent extraction and mass balance were similar between 220–440 mL/min at 30 h of operation (Figure 3b), a moderate flow rate of 220 mL/min was chosen for conducting the experiments in the next sections.

### 3.4. Effect of the NaCl Concentration in the Receiver Compartment

**Figure 4:**
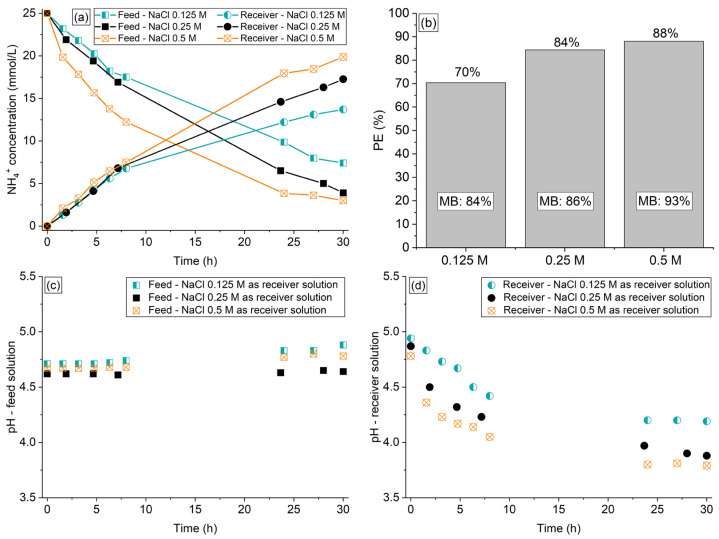
(**a**) NH4^+^ concentration profile, (**b**) percent extraction, mass balance, (**c**) pH profile of the synthetic feed, and (**d**) receiver solutions of the experiments conducted with NaCl 0.125 M, 0.25 M, and 0.5 M as the receiver solution.

The influence of NaCl concentration in the receiver compartment was evaluated for 30 h using solutions of 0.125 M, 0.25 M, and 0.5 M (presenting Na^+^ (receiver)/NH_4_^+^ (feed) molar ratios of 5, 10, and 20, respectively). The organic acids and phosphate ions remained in the feed solution. The results of ammonium concentration are shown in Figure 4a, whereas the NH_4_^+^ percent extraction and mass balance are shown in Figure 4b. The pH vs. time profiles of the solutions are shown in Figure 4c,d.

As expected, increasing the NaCl concentration between 0.125 M and 0.5 M enhanced considerably the degree of ammonium transfer, since the concentration of driving Na^+^ counter-ions across the membrane increased. Similar results were obtained by Wang et al. [19] using H^+^ as driving counter-ions. 

One can note in Figure 4a that the NH_4_^+^ concentration in the receiver compartment did not increase following the same proportion of the reduction in the feed compartment for all tests. According to Figure 4b and Table S5, as the NaCl concentration was increased, the liquid phase mass balance of NH_4_^+^ also increased, becoming closer to 100%. This indicates that the NH_4_^+^ sorption in the membrane was reduced with increasing NaCl concentration (final mass balance of 84%, 86%, and 91% with NaCl receiver solutions at 0.125 M, 0.25 M, and 0.5 M, respectively). Besides, the NaCl concentration slightly affected the water transfer from the feed to the receiver solution, since this phenomenon was more intense with a NaCl solution at 0.5 M, showing a volume deviation of 4%.

According to Figure 4c,d, the pH profiles of the solutions showed a similar behavior for all NaCl concentrations tested: an increase for the feed solution and a reduction for the receiver one, as discussed in the previous sections. The intensity of the pH increase/decrease was similar for the three NaCl concentrations, although the NH_4_^+^ transfer occurred more intensely with increasing NaCl concentration (Figure 4a). 

Considering the relatively low NH_4_^+^ transfer and its intense sorption observed with the 0.125 M NaCl solution, the use of this receiver solution must be avoided. Therefore, the NaCl solutions at 0.25 M and 0.5 M were used in the experiments described in the next section to evaluate the influence of the volume ratio of the feed:receiver solutions.

### 3.5. Effect of Volume Ratio of Feed:Receiver Solutions

**Figure 5:**
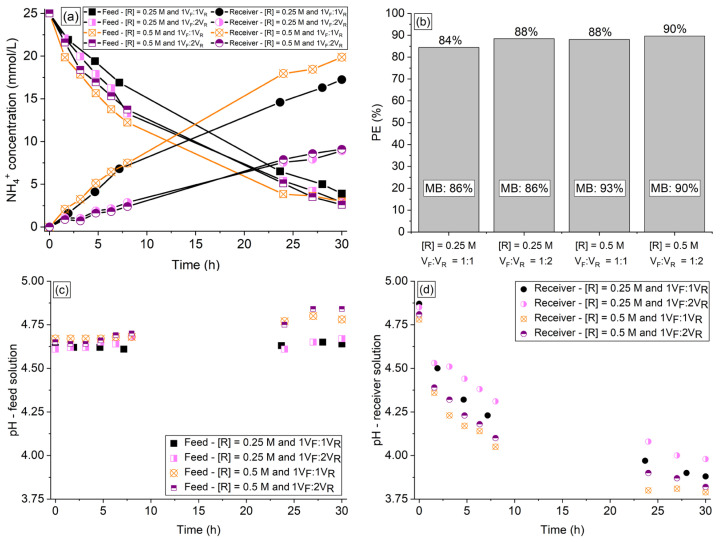
(**a**) NH4^+^ concentration profile, (**b**) percent extraction, mass balance, (**c**) pH profile of the synthetic feed, and (**d**) receiver solutions of the experiments conducted using receiver concentration ([R]) of 0.25 M and 0.5 M, and feed:receiver volume ratio of 1:1 and 1:2.

The effect of the volume ratio of feed:receiver solutions was evaluated for 30 h. The organic acids and phosphate ions remained in the feed solution. Figure 5a shows the NH_4_^+^ concentration time course in the two compartments, Figure 5b shows the NH_4_^+^ percent extraction and mass balance, and Figure 5c,d shows the pH vs. time profiles of the solutions from the tests conducted under the following conditions of receiver concentration ([R]) and volume ratio of feed:receiver solutions: (1) [R] = 0.25 M and V_F_:V_R_ = 1:1; (2) [R] = 0.25 M and V_F_:V_R_ = 1:2; (3) [R] = 0.5 M and V_F_:V_R_ = 1:1; (4) [R] = 0.5 M and V_F_:V_R_ = 1:2. 

According to Figure 5b, increasing V_F_:V_R_ from 1:1 to 1:2 increased the NH_4_^+^ extraction degree with the receiver solution at 0.25 M from 84% to 88%, whereas for the system with the receiver solution at 0.5 M, the NH_4_^+^ extraction degree increased from 88% to 90%, respectively. Note in Figure 5b that the final percent extraction obtained for V_F_:V_R_ of 1:2 using receiver concentration of 0.25 M was similar to that obtained for V_F_:V_R_ of 1:1 with a receiver concentration of 0.5 M (88%), although the NH_4_^+^ concentration profiles of the feed solutions showed different behaviors in the first hours. As expected, the final ammonium concentration in the receiver solution using 1V_F_:2V_R_ was equal to approximately the half of the concentration obtained using 1V_F_:1V_R_.

From the perspective of the PHA production process, several factors, such as the organic loading rate, the desired carbon to nitrogen (C:N) molar ratio, the number of F/f cycles per day, and the hydraulic retention time, influence the amount of ammonium required to be added to the bioreactor for each F/f cycle. Higher ammonium concentrations in the receiver solution could facilitate ammonium recovery as a nutrient during the culture selection stage, as the ammonium-rich solution (receiver) can be added to the bioreactor in lower volumes to achieve the desired C:N ratio. In cases where the concentration of ammonium in the receiver solution is much lower than what is required for the production process, the receiver solutions can be reused until the minimal concentration required for the Donnan dialysis process is reached, since the ratio between the driving (Na^+^) counter-ions in the used receiver solution and target (NH_4_^+^) counter-ion in the new feed solution remains high enough to allow for a subsequent DD batch.

The volume deviation (in relation to the expected value) when V_F_:V_R_ was increased from 1:1 to 1:2 with the receiver solution at 0.25 M remained below 3%, whereas the volume deviation for the receiver solution at 0.5 M and both V_F_:V_R_ ratios remained at around 4%, which occurred due to the increase in osmotic pressure difference between the diluted and concentrated solutions, leading to water transfer to the receiver compartment.

According to Figure 5c, increasing V_F_:V_R_ from 1:1 to 1:2 for both receiver solution concentrations (0.25 M and 0.5 M) caused virtually no change in the pH profile of the feed solution, although a slight increase in NH_4_^+^ extraction was observed with increasing V_F_:V_R_, especially with the receiver solution at 0.25 M (Figure 5a,b). On the other hand, the volume ratio affected considerably the pH of the receiver solution (Figure 5d). Note that for both receiver concentrations (0.25 M and 0.5 M), the pH reduction in the receiver compartment was considerably less intense for V_F_:V_R_ of 1:2 than for 1:1, although the NH_4_^+^ extraction was slightly greater with V_F_:V_R_ of 1:2. This was expected since the concentration of NH_4_^+^ ions was lower in the receiver solution with a larger volume. 

As shown in Figure 5b and Table S6, the increase in V_F_:V_R_ from 1:1 to 1:2 caused practically no effect on the NH_4_^+^ mass balance for both receiver concentrations, whereas increasing the NaCl concentration enhanced the mass balance for both V_F_:V_R_ ratios. This supports the previous discussion on the greater desorption of NH_4_^+^ ions from the membrane during the DD operation at higher concentrations of Na^+^ in the receiver solution. On the other hand, high concentrations of Na^+^ ions in the final/treated feed solution, such as the one achieved in the system with the receiver solution at 0.5 mol/L and V_F_:V_R_ of 1:2 (maximum of 23 g sodium/L in the final/treated feed solution), might limit its use in PHA production systems with halotolerant PHA-accumulating organisms. Therefore, to allow the use of the final/treated feed solution in a wider range of PHA production systems, the experiments of the next section (Section 3.6) were conducted using NaCl solution at 0.25 M and V_F_:V_R_ of 1:2 (maximum of 11.5 g sodium/L in the final/treated feed solution). In Section 3.6, the application of an external electric potential to the system was evaluated as a method to intensify the ammonium transfer without hindering the ammonium mass balance.

### 3.6. Evaluation of External Electric Potential Application to the Two-Compartment System

The application of external electric potential difference to the two-compartment system for intensifying the NH_4_^+^ transfer was tested. The electrode present at the receiver solution compartment was the cathode, while the electrode at the feed solution compartment was the anode. This configuration was chosen because although Na^+^ ions of the receiver solution were attracted to the cathode, their concentration (0.25 M) was 10 times higher than the concentration of ammonium ions (0.025 M) in the feed solution. Therefore, under potential application, NH_4_^+^ ions tended to migrate from the feed to the receiver solution due to both the difference in their concentration and the electric potential applied, whereas the Na^+^ ions tended to migrate from the receiver to the feed solution due to the difference in their concentration.

#### 3.6.1. Current–Voltage Curves of Membrane/Electrolytes Systems

**Figure 6:**
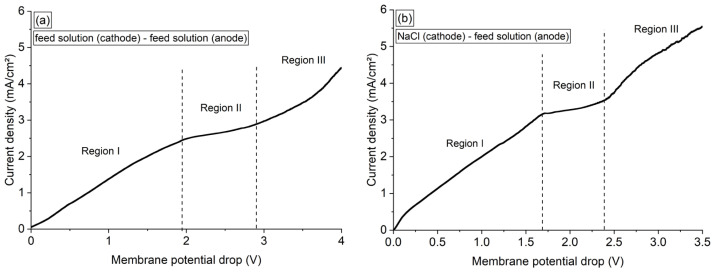
Current–voltage curves of the membrane/solution system obtained using the following configurations: (**a**) synthetic feed solution (cathode)—synthetic feed solution (anode), (**b**) NaCl 0.25 M (cathode)—synthetic feed solution (anode).

The current–voltage curves shown in Figure 6 were constructed by linear sweep voltammetry using Ralex membrane and two configurations regarding the solutions present in each compartment: (a) synthetic feed—synthetic feed, and (b) NaCl 0.25 M—synthetic feed solutions. Note that the curves presented three well-defined regions. In region I, also called the quasi-ohmic region, ion transport is governed by diffusion–migration. Region II is a transition zone between regions I and III, and the range of membrane potential drop of this region (plateau length) is related to the energy required to change the dominant ion transport mechanism from diffusion–migration to over-limiting mechanisms. In region III, over-limiting mechanisms of mass transfer, such as electroconvection, gravitational convection, and water dissociation, become dominant [20,41].

From Figure 6a, the limiting current density, ohmic resistance, and plateau length of the membrane/electrolyte in the feed–feed solutions system were determined as 2.4 mA/cm^2^, 749 Ω·cm^2^, and 1.1 V, respectively. The limiting current density of this system was related to the depletion of NH_4_^+^ in the diffusion boundary layer during concentration polarization since these were the main cationic species (counter-ions) present in solution. 

From Figure 6b, the limiting current density (3.2 mA/cm^2^), ohmic resistance (586 Ω·cm^2^), and plateau length (0.6 V) of the NaCl–feed system were determined, which was the configuration used in the DD operations. Note that the limiting current density of the NaCl–feed system was higher than that of the feed–feed system, whereas the ohmic resistance and plateau length of the former were lower than that of the latter. In the case of the NaCl–feed system, the solutions on each side of the membrane were different, with Na^+^ concentration 10 times greater than NH_4_^+^. Therefore, the Na^+^ ions present in the cathode compartment migrated through the membrane towards the anode due to the difference in concentration of the solutions, while the NH_4_^+^ ions present in the anode compartment migrated to the cathode compartment due to the difference in concentration and electric potential. Thus, in addition to the NH_4_^+^ ions migrating through the membrane towards the cathode, there were also Na^+^ ions reaching the liquid diffusion boundary layer at the membrane–feed solution interface. This transport of NH_4_^+^ and Na^+^ ions in opposite directions explains the difference in the values of limiting current density of the feed–feed and NaCl–feed systems. The ohmic resistance and plateau length were lower for the NaCl-feed system because of the higher concentration of Na^+^ ions than NH_4_^+^ and the higher conductivity of the NaCl solution than the feed solution [30]. Lastly, the potential at which concentration polarization began to occur strongly in the NaCl–feed system was 1.7 V. Thus, under all potentials applied to the two-compartment system (−0.1 V, −0.6 V, and −1 V) in Section 3.6.2., the membrane operated in the quasi-ohmic condition. In this case, the membrane system was not affected by the limitations related to the intense occurrence of concentration polarization, such as water dissociation at the membrane and fouling/scaling phenomena.

#### 3.6.2. Evaluation of NH_4_^+^ Transfer under External Electric Potential Application

**Figure 7:**
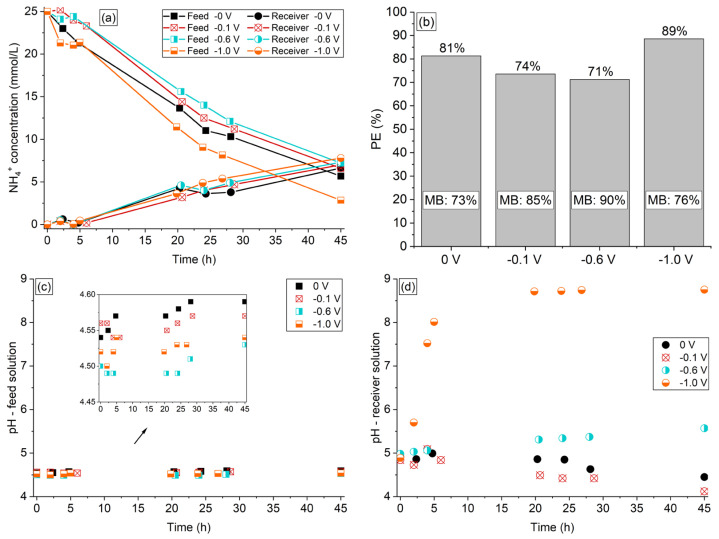
(**a**) NH4^+^ concentration profile, (**b**) percent extraction, mass balance, (**c**) pH profile of the synthetic feed, and (**d**) receiver solutions of the experiments conducted under application of 0 V, −0.1 V, −0.6 V, and −1.0 V.

The application of electric potential difference of −0.1 V, −0.6 V, and −1 V to the two-compartment system was evaluated as described in Section 2.2. As the experiments were conducted in an electrochemical cell different from the one used in the previous DD experiments, and the ratio of membrane area/volume of solutions was reduced, the tests were conducted for 45 h. The organic acids and phosphate ions remained in the feed solution. The curves of NH_4_^+^ concentration time course profiles are shown in Figure 7a, the NH_4_^+^ percent extraction and mass balance are shown in Figure 7b, and the pH vs. time profiles are shown in Figure 7c,d.

According to Figure 7a, the external potential application from 0 to −1 V did not alter considerably the concentration of NH_4_^+^ ions reaching the receiver compartment since the curves are relatively close. This indicates that the driving force from the potential application acting in the opposite direction to the driving force from the concentration difference in the compartments did not considerably affect the rate of NH_4_^+^ ions entering into the receiver compartment. Regarding the feed compartment, increasing the applied potential from 0 to −0.6 V impaired the ammonium extraction (Figure 7b), since a greater amount of ions remained in the feed solution. On the other hand, as shown in Figure 7b and Table S7, increasing the potential between 0 and −0.6 V improved the ammonium mass balance (73% for 0 V and 90% for −0.6 V). This indicates that the application of a potential up to −0.6 V mitigated the sorption of NH_4_^+^ in the membrane or favored their desorption, which could eliminate the chemical regeneration step of the membrane after each batch. According to Figure 7a, the application of the highest potential (−1 V) considerably reduced the NH_4_^+^ concentration in the feed compartment, but this decrease did not occur in the same proportion as the increase in the receiver compartment, causing a considerable reduction in the ammonium mass balance (76%). This behavior can be explained by the occurrence of intense hydrogen evolution reaction (HER) at the cathode, since in this case the two-compartment system operated as a membrane electrolysis process, and the consequent pH alterations. According to the speciation diagram constructed for ammonia/ammonium ions with the aid of Hydra–Medusa software (v.1., Royal Institute of Technology, Stockholm, Sweden) [42] (Figure S2), the gaseous NH_3_ begins to be formed at pH 7 and reaches the same concentration as that of NH_4_^+^ at pH 9.2. In this sense, the intense consumption of protons from the solution during HER at the cathode caused an increase in the bulk solution pH [43], thus converting NH_4_^+^ ions into gaseous NH_3_. As the electrochemical system was not sealed, the gaseous species migrated to the atmosphere, causing a reduction in the mass balance. 

According to the pH profiles of the solutions shown in Figure 7c,d, the pH of the receiver solution reached approximately 8.5 after 20 h of operation under −1 V, which supports the above-mentioned discussion on the NH_3_ formation. It is worth mentioning that the pH at the electrode surface must have reached even higher values than the bulk solution during HER. Note in Figure 7d that the intense pH increase of the receiver solution occurred only under −1 V, indicating that although HER also occurred at lower potentials, such as −0.6 V, the consumption of protons was not intense enough to increase the solution pH strongly. For the systems under 0 V and −0.1 V, the typical reduction in pH was verified for the receiver solution, which occurred because of the transferred NH_4_^+^ ions from the feed solution, as discussed in previous sections. The pH of the feed solution (Figure 7c) showed a practically constant behavior throughout the experiments, showing a slight increase mainly in longer times. An increase in the feed compartment pH was expected due to the transfer of NH_4_^+^ ions to the receiver solution. On the other hand, it is known that H^+^ ions are formed at the anode, which was located in the feed compartment thus reducing the solution pH. Most probably, these two opposing pH effects led to the practically constant pH behavior of the feed solution.

The external electric potential application intensified the occurrence of water transfer through the membrane, which may have occurred by electroosmosis in addition to osmosis. When electroosmosis takes place in membrane systems, ions passing through the membrane are accompanied by a shell solvation of water molecules [33], which occurs more intensively under application of low current densities [44,45]. Indeed, the receiver solution volume deviation in relation to the expected value was higher at −0.1 V (6%) than at −1 V (4.8%). For the experiment conducted without external electric potential application, the volume deviation was below 3%. It is known that the water transfer phenomena can limit the usefulness of membrane separation processes as a method for concentrating electrolyte solutions [46]. The results presented here indicate that the application of an external electric potential to the two-compartment system using the Ralex membrane did not result in an improved ammonium separation degree. Thus, the experiments using real solutions of the next section were conducted without external electric potential application. 

### 3.7. Evaluation of DD Application for Treating a Real Solution from a Fermentation Process

**Figure 8:**
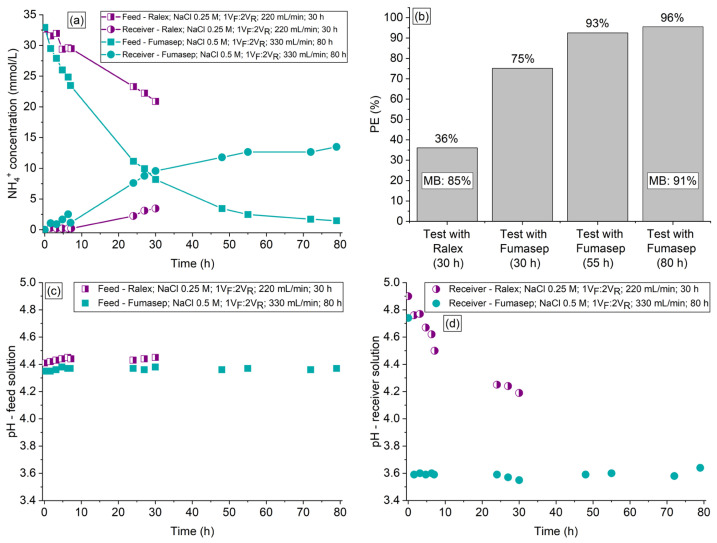
(**a**) NH4^+^ concentration profile, (**b**) percent extraction, mass balance, (**c**) pH profile of the feed, and (**d**) receiver solutions of the experiments conducted with a real feed solution using Ralex and Fumasep membranes applying different operational conditions.

Based on the results obtained in the previous experiments conducted with the synthetic feed solution, an experiment was carried out using a real feed solution from the acidogenic fermentation of cheese whey using Ralex membrane and applying operating conditions optimized in the previous sections (NaCl at 0.25 M as receiver solution, V_F_:V_R_ of 1:2, flow rate of 220 mL/min, and duration of 30 h). The ammonium concentration profiles observed in the two compartments are shown in Figure 8a, the NH_4_^+^ percent extraction and mass balance are shown in Figure 8b, and the pH vs. time profiles are shown in Figure 8c,d. It is important to note that the composition of the real solution evaluated in this section is different from the composition of the synthetic solution used in the previous sections, mainly in terms of the VFAs contents (Table 3 and Table 4). This difference in the compositions occurred because of variations in the operational conditions of the acidogenic fermentation of cheese whey, which generated feed solutions more concentrated in ammonium and VFAs than expected.

As expected, the use of a real feed solution in the DD system with the Ralex membrane altered the ammonium concentration vs. time profile when compared to the tests conducted with synthetic solutions. Note in Figure 8b that the ammonium percent extraction after 30 h was 36%, which is a value considerably lower than the one obtained with the synthetic solution under the same operating conditions (88%—Figure 5b). According to Figure 5b and Figure 8b, the mass balance was not affected by the type of feed solution (~85%). This reduction in the degree of NH_4_^+^ recovery can be explained by the higher complexity of the real feed solution than the synthetic one, which presented greater conductivity (7.9 mS/cm^2^ and 3.9 mS/cm^2^, respectively), concentration of ammonium (33 Nmmol/L and 25 Nmmol/L, respectively), and other cationic species (Table 4). The considerably greater Cmol:Nmol ratio in the real solution (8.5:1) than that in the synthetic one (1:1), besides the presence of anionic (co-ions) species shown in Table 4, affected the process performance, and it was not possible to directly translate the optimal parameters obtained for the model solutions to those required for the real solution. Therefore, we performed additional tests in order to determine the optimal parameters for the real solution, which were obtained under operating conditions that we refer to herein as “stronger” conditions (Fumasep membrane, NaCl at 0.5 M as receiver solution, V_F_:V_R_ of 1:2, flow rate of 330 mL/min, and duration of 80 h), and the results obtained are shown in Figure 8a–d. For the experiment conducted under “stronger conditions”, Figure 8b presents the NH_4_^+^ percent extraction obtained at three different times: after 30 h (the same as the test conducted with Ralex membrane), 55 h (beginning of steady state condition), and 80 h (end of experiment). Note in Figure 8b and Table S8 that, in this case, the PE% increased to 75% after 30 h of experiment, 93% after 55 h, and 96% after 80 h, whereas the final mass balance increased to 91%. Although the experiment had a duration of 80 h, only 55 h would be sufficient since the process achieved a quasi-steady state condition. The Cmol:Nmol ratio obtained after 55 and 80 h of DD operation were 112:1 and 186:1, respectively (the initial one was 8.5:1), which means that the PHA production process can be carried out under controlled conditions of ammonium availability and, therefore, allowing to subject the culture to an uncoupled C:N feeding strategy during culture selection and ammonium-limitation during PHA production. The concentration of trace elements and other cationic and anionic species present in the initial and final real feed and receiver solutions is shown in Table S9. Note that some cationic species were transported from the feed compartment to the receiver one, mainly those present at the highest concentrations, such as calcium, potassium, magnesium, and zinc, as expected. According to the table, there was no significant transport of anionic species (co-ions) through the membranes. The organic acids and phosphate ions remained in the real feed solution for both tests conducted with the real feed solution. As verified for the tests carried out with synthetic solutions, the use of Fumasep with the real feed solution under “stronger” conditions intensified the occurrence of water transfer through the membrane when compared to the test conducted with the Ralex membrane under “milder” conditions (volume deviations of 4% and 2% in relation to the expected value, respectively).

These results imply that the ammonium removal from the fermented cheese whey will restrict the cellular growth during the PHA production as well as will improve the culture selection with PHA-accumulating organism through the uncoupling of carbon and nitrogen availabilities, improving the PHA productivity and process performance. In that way, the high removal of ammonium obtained, resulting in a high C:N ratio, will impact positively the PHA production process as an uncoupled C:N strategy can be applied. 

The pH profiles of the solutions presented in Figure 8c,d show that the pH variation in the feed solution was very subtle, showing a practically constant behavior throughout both tests. On the other hand, the receiver solution showed a strong reduction in pH, especially for the test conducted with the Fumasep membrane. This behavior for Fumasep was also verified with the synthetic solution (Section 3.1.1) and is in accordance with the intense transfer of ammonium through this membrane in the first hours of the test (Figure 8a). Despite the intense transfer of NH_4_^+^ through Fumasep for approximately 50 h, the pH of the receiver solution did not show significant changes after the sharp drop that occurred in the first 2 h, which supports the discussions presented in the previous sections. For the tests carried out with real solutions, the pH effect was more notable in the receiver solution because it was composed of a neutral salt, whereas the feed solution was a complex mixture having pH buffering properties.

## 4. Conclusions

- The feasibility of using Donnan dialysis under batch operating conditions to separate ammonium ions from volatile fatty acids present in synthetic and real solutions to be used as feedstock in the PHA production process was evaluated using three commercial cation-exchange membranes with distinct properties.
- The membrane type influenced strongly the NH_4_^+^ sorption and membrane mass transfer. Fumasep and Ralex membranes showed to be the most appropriate membranes for the process, whereas the use of the Ionsep membrane for the specific purpose evaluated herein should be avoided.
- The ammonium transfer rates using HCl and NaCl as receiver solution were very similar except for the NH_4_^+^ sorption effect, which was more intense with NaCl. Although sorption might reduce the membrane ion-exchange capacity in long-term operations, the overall ammonium recovery can be increased if a desorption (regeneration) step is carried out after each DD batch operation.
- Higher volumetric flow rates initially increased the NH_4_^+^ transfer, but the final degrees of NH_4_^+^ recovery were very close for flow rates of 220 to 440 mL/min, corresponding to Reynolds numbers of 410 to 820, respectively. The increase in NaCl concentration of the receiver solution enhanced considerably the NH_4_^+^ extraction.
- The increase in V_F_:V_R_ from 1:1 to 1:2 maintaining the NaCl concentration at 0.25 M generated a NH_4_^+^ percent extraction very similar to that obtained with V_F_:V_R_ of 1:1 and concentration of 0.5 M NaCl.
- An external electric potential application to the two-compartment Donnan dialysis system used hindered the NH_4_^+^ transfer rate but favored its desorption from the membrane. A possible strategy is to apply an electric potential difference between −0.1 V and −0.6 V at the end of the process in order to eliminate or facilitate the chemical regeneration step of the membrane into its Na^+^ form.
- When treating the real solution obtained from the acidogenic fermentation of cheese whey, C:N molar ratios of 112:1 and 186:1 were obtained after 55 and 80 h, respectively, of DD operation under the following optimized conditions: Fumasep membrane with NaCl at 0.5 M as receiver solution, feed:receiver volume ratio of 1:2, and solutions flow rate of 330 mL/min.

Our ongoing work includes evaluation of the Donnan dialysis process potential for a simultaneous separation and recovery of ammonium and phosphate ions since the latter is also an important nutrient used in the PHAs bioproduction.

## Figures and Tables

**Table 1:** Experimental conditions of the Donnan dialysis experiments.

Evaluation	Membrane	Feed Solution	Receiver Solution	Flow Rate (mL/min)	Volume Ratio of Feed: Receiver	Potential Applied (V)
Membrane type	Fumasep, Ralex, Ionsep	Synthetic	NaCl 0.25 M	220	1:1	No
Receiver solution type	Ralex	Synthetic	NaCl 0.25 M, HCl 0.25 M	220	1:1	No
Flow rate	Ralex	Synthetic	NaCl 0.25 M	110, 220, 330, and 440	1:1	No
Receiver solution concentration	Ralex	Synthetic	NaCl 0.125 M, 0.25 M, 0.5 M	220	1:1	No
Volume ratio of feed:receiver solutions	Ralex	Synthetic	NaCl 0.25 M and 0.5 M	220	1:1, 1:2	No
External electric potential application	Ralex	Synthetic	NaCl 0.25 M	220	1:2	No, −0.1, −0.6, −1.0
Operation with a real feed solution	Ralex, Fumasep	Real	NaCl 0.25 M and 0.5 M	220, 330	1:2	No

**Table 2:** Main characteristics of the investigated membranes.

	Ralex CMHPES [22,23]	IONSEP-HC-C [24,25]	Fumasep FKS-PET-130 [26,27]
Type	Heterogeneous	Heterogeneous	Homogeneous
Ion-exchange group	R–SO_3_^−^	R–SO_3_^−^	Not specified
Thickness of dry membrane [μm]	450	450	90–100
Thickness of swelled membrane [μm]	700	700	127
pH stability	0–10	Not specified	0–8
Ion-exchange capacity [meq/g]	2.2	2.0	0.74
Water content [%]	55	35–50	19

**Table 3:** Chemical composition of the synthetic feed solution.

	Component	Concentration
Nutrients	NH_4_Cl—N	25 Nmmol/L
	KH_2_PO_4_—P	1.1 Pmmol/L
VFAs	Acetic acid	6.18 Cmmol/L
	Propionic acid	6.24 Cmmol/L
	Butyric acid	6.32 Cmmol/L
	Valeric acid	6.20 Cmmol/L
Trace elements	Copper	0.00054 mmol/L
	Iron	0.00081 mmol/L
	Molybdenum	0.00055 mmol/L
	Nickel	0.04104 mmol/L

**Table 4:** Chemical composition of the real feed solution.

	Component	Concentration
Solids	TSS *	0.55 ± 0.00 g/L
	VSS **	0.48 ± 0.07 g/L
Nutrients	N-NH_4_	33 Nmmol/L
	P-PO_4_	2.2 Pmmol/L
VFAs	Acetic acid	109.08 Cmmol/L
	Propionic acid	34.56 Cmmol/L
	Butyric acid	132.97 Cmmol/L
	Valeric acid	2.98 Cmmol/L
Trace/other cationic species	Aluminium	0.006 mmol/L
	Boron	0.014 mmol/L
	Calcium	3.558 mmol/L
	Copper	0.002 mmol/L
	Iron	0.008 mmol/L
	Magnesium	1.495 mmol/L
	Potassium	12.39 mmol/L
	Silicon	0.120 mmol/L
	Sodium	27.65 mmol/L
	Strontium	0.005 mmol/L
	Zinc	0.032 mmol/L
Trace/other anionic species	Bromide	0.027 mmol/L
	Chloride	42.02 mmol/L
	Fluoride	23.38 mmol/L
	Nitrate	0.025 mmol/L
	Nitrite	2.042 mmol/L
	Sulfate	0.447 mmol/L

* Total suspended solids; ** Volatile suspended solids.

## Data Availability

Not applicable.

## Supplementary Materials

The following supporting information can be downloaded at: https://www.mdpi.com/article/10.3390/membranes13030347/s1, Equations (S1)–(S5): Calculation of the theoretical and real/final mass balances of ammonium ions. Table S1: Theoretical and real mass balance (%) of ammonium ions present in the synthetic feed and receiver solutions of the experiments conducted with Fumasep, Ralex, and Ionsep membranes (Figure 1); Table S2: Ammonium concentration values in the synthetic feed and NaCl/HCl solutions obtained in the evaluation of ammonium sorption occurrence in Fumasep, Ralex and Ionsep membranes. Table S3: Theoretical and real mass balance (%) of ammonium ions present in the synthetic feed and receiver solutions of the experiments conducted HCl and NaCl as receiver solution (Figure 2); Table S4: Theoretical and real mass balance (%) of ammonium ions present in the synthetic feed and receiver solutions of the experiments conducted using flow rate of 110, 220, 330, and 440 mL/min (Figure 3); Table S5: Theoretical and real mass balance (%) of ammonium ions present in the synthetic feed and receiver solutions of the experiments conducted with NaCl solutions at 0.125 M, 0.25 M, and 0.5 M (Figure 4); Table S6: Theoretical and real mass balance (%) of ammonium ions present in the synthetic feed and receiver solutions of the experiments conducted with NaCl solutions ([R]) at 0.25 M and 0.5 M, and feed:receiver volume ratio (V_F_:V_R_) of 1:1 and 1:2 (Figure 5); Table S7: Theoretical and real mass balance (%) of ammonium ions present in the synthetic feed and receiver solutions of the experiments conducted under electric potential application of 0 V, −0.1 V, −0.6 V, and −1.0 V (Figure 7).; Table S8: Theoretical and real mass balance (%) of ammonium ions present in the feed and receiver solutions of the experiments conducted with a real feed solution using Ralex and Fumasep membranes at different conditions (Figure 8); Table S9: Molar concentration (mmol/L) of trace/other cationic and anionic species present in the initial and final real feed and receiver solutions.; Section S1: Membrane characterization by means of ATR-FTIR.; Figure S1: IR spectra of Fumasep, Ralex and Ionsep membranes.; Figure S2: Speciation diagram for ammonia/ammonium ions. References [47,48,49,50,51,52,53,54,55,56,57,58,59,60,61,62,63,64,65,66,67,68,69,70,71,72,73,74] are cited in Supplementary Materials.
