# Supplementary material for: Donnan Dialysis for Recovering Ammonium from Fermentation Solutions Rich in Volatile Fatty Acids
Source: Membranes (Basel). 2023 Mar 17;13(3):347. doi: 10.3390/membranes13030347 (PMC10054700; doi:10.3390/membranes13030347)
Supplement: Supplementary file 1 [file membranes-13-00347-s001.zip › membranes-2286521-supplementary.pdf]

Supplementary Materials

Donnan Dialysis for Recovering Ammonium from Fermentation Solutions Rich in Volatile Fatty Acids

This supplementary material presents:

(1) Equations S1–S5, which were used to calculate the theoretical and real/final mass balances of ammonium ions.
(2) Tables S1, S3–S8, which contain the results of theoretical and real/final mass balance of ammonium ions present in the feed and receiver solutions after each Donnan dialysis experiment.
(3) Table S2, which presents the results of the evaluation of ammonium sorption occurrence in Fumasep, Ralex and Ionsep membranes.
(4) Table S9, which contains the molar concentration (mmol/L) of trace/other cationic and anionic species present in the initial and final real feed and receiver solutions.
(5) Section S1 and Figure S1, which present the results of membrane characterization by means of ATR-FTIR.
(6) Figure S2, which shows the speciation diagram for ammonia/ammonium ions.

Equations S1 – S5: Calculation of the theoretical and real/final mass balances of ammonium ions

Equations S1 – S3 were used to calculate the theoretical mass balance of ammonium ions at different operating times (*i*), whereas Equations S2, S4 and S5 were used to calculate the real/final mass balance. In the equations, the subscripts *F* and *R* represent feed and receiver solutions, respectively, and the subscripts 0, *i* and *f* represent initial state, time *i* and final time (end of experiment), respectively.

$$\text{Theoretical mass balance}_{\text{i}} = 100 + \left(\frac{\text{Theoretical } NH_4^+ \text{ mass}_{(\text{F+R+aliquots})_{\text{i}}} - NH_4^+ \text{ mass}_{(\text{F+R})_0}}{NH_4^+ \text{ mass}_{(\text{F+R})_0}}\right) \quad \text{(S1)}$$

$$NH_4^+\ mass_{(F+R)_0} = C_{F_0}V_{F_0} + C_{R_0}V_{R_0} \quad \text{(S2)}$$

$$Theoretical\ NH_4^+\ mass_{(F+R+aliquots)_i} = \left(C_{F_i} \times theoretical\ V_{F_i}\right) + \left(C_{R_i} \times theoretical\ V_{R_i}\right) + \sum_{i=0}^{i}\left[C_{(aliquots\ of\ F)_i} \times V_{(aliquots\ of\ F)_i}\right] + \sum_{i=0}^{i}\left[C_{(aliquots\ of\ R)_i} \times V_{(aliquots\ of\ R)_i}\right] \quad \text{(S3)}$$

$$Real/final\ mass\ balance = 100 + \left(\frac{Real/final\ NH_4^+\ mass_{(F+R+aliquots)_f} - NH_4^+\ mass_{(F+R)_0}}{NH_4^+\ mass_{(F+R)_0}}\right) \quad \text{(S4)}$$

$$Real/final\ NH_4^+\ mass_{(F+R+aliquots)_f} = \left(C_{F_f} \times real\ V_{F_f}\right) + \left(C_{R_f} \times real\ V_{R_f}\right) + \sum_{i=0}^{f}\left[C_{(aliquots\ of\ F)_i} \times V_{(aliquots\ of\ F)_i}\right] + \sum_{i=0}^{f}\left[C_{(aliquots\ of\ R)_i} \times V_{(aliquots\ of\ R)_i}\right] \quad (S5)$$

Table S1. Theoretical and real mass balance (%) of ammonium ions present in the synthetic feed and receiver solutions of the experiments conducted with Fumasep, Ralex, and Ionsep membranes (Figure 1).

	Theoretical Mass Balance (%)		
Aliquot	Fumasep	Ralex	Ionsep
1	100	100	100
2	96	93	89
3	94	93	83
4	98	94	86
5	99	58	79
6	99	84	82
7	99	83	80
8	100	85	79
9	98	84	78
10	98	84	80
11	97	84	79
12	96	86	81
13	96		81
14	97		
	Real/final mass balance (%)		
	100	86	84

Table S2. Ammonium concentration values in the synthetic feed and NaCl/HCl solutions obtained in the evaluation of ammonium sorption occurrence in Fumasep, Ralex and Ionsep membranes.

Test conducted with NaCl			
	Fumasep	Ralex	Ionsep
Ammonium concentration in the initial feed solution (mmol/L)		24.7	
Ammonium concentration in the final feed solution (mmol/L)	24.7	19.4	18.4
Ammonium sorbed per dry membrane mass (mmol/g)	0	1.3	1.2
Ammonium concentration in the final NaCl solution (mmol/L)	0.9	4.4	4.4
Final mass balance (%)	103.5	96.1	92.0
Test conducted with HCl			
	Fumasep	Ralex	Ionsep
Ammonium concentration in the initial feed solution (mmol/L)		24.7	
Ammonium concentration in the final feed solution (mmol/L)	25.6	19.7	19.6
Ammonium sorbed per dry membrane mass (mmol/g)	0	1.2	1.2
Ammonium concentration in the final NaCl solution (mmol/L)	0.8	4.5	4.5
Final mass balance (%)	103.6	97.7	97.1

Table S3. Theoretical and real mass balance (%) of ammonium ions present in the synthetic feed and receiver solutions of the experiments conducted HCl and NaCl as receiver solution (Figure 2).

	Theoretical mass balance (%)	
Aliquot	HCl	NaCl
1	100	100
2	92	93
3	89	93
4	95	94
5	91	58
6	89	84
7	95	83
8	93	85
9	93	84
10	93	84
11	95	84
12	93	86
13	96	
	Real/final mass balance (%)	
	97	86

Table S4. Theoretical and real mass balance (%) of ammonium ions present in the synthetic feed and receiver solutions of the experiments conducted using flow rate of 110, 220, 330, and 440 ml/min (Figure 3).

	Theoretical mass balance (%)			
Aliquot	110 mL/min	220 mL/min	330 mL/min	440 mL/min
1	100	100	100	100
2	95	93	89	90
3	88	93	87	81
4	88	94	88	84
5	84	58	80	79
6	83	84	77	80
7	90	83	88	81
8	89	85	85	84
9	91	84	85	86
	Real/final mass balance (%)			
	92	86	87	88

Table S5. Theoretical and real mass balance (%) of ammonium ions present in the synthetic feed and receiver solutions of the experiments conducted with NaCl solutions at 0.125 M, 0.25 M, and 0.5 M (Figure 4).

Aliquot	Theoretical mass balance (%)		
	0.125 M	0.25 M	0.5 M
1	100	100	100
2	97	93	87
3	97	93	83
4	97	94	82
5	94	58	75
6	96	84	78
7	87	83	86
8	84	85	78
9	84	84	90
	Real/final mass balance (%)		
	84	86	93

Table S6. Theoretical and real mass balance (%) of ammonium ions present in the synthetic feed and receiver solutions of the experiments conducted with NaCl solutions ([R]) at 0.25 M and 0.5 M, and feed:receiver volume ratio (V_F:V_R) of 1:1 and 1:2 (Figure 5).

Aliquot	Theoretical mass balance (%)			
	[R] = 0.25 M and V_F:V_R = 1:1.	[R] = 0.25 M and V_F:V_R = 1:2.	[R] = 0.5 M and V_F:V_R = 1:1.	[R] = 0.5 M and V_F:V_R = 1:2.
1	100	100	100	100
2	93	96	87	93
3	93	87	83	80
4	94	86	82	82
5	58	81	75	78
6	84	76	78	77
7	83	83	86	87
8	85	81	78	87
9	84	84	90	88
	Real/final mass balance (%)			
	86	86	92	90

Table S7. Theoretical and real mass balance (%) of ammonium ions present in the synthetic feed and receiver solutions of the experiments conducted under electric potential application of 0 V, −0.1 V, −0.6 V, and −1.0 V (Figure 7).

Aliquot	Theoretical mass balance (%)			
	0 V	−0.1 V	−0.6 V	−1.0 V
1	100	100	100	100
2	97	101	99	87
3	87	94	96	83
4	89	93	98	88
5	74	83	88	75
6	73	82	88	76
7	78	83	88	76
8		84		76
	Real/final mass balance (%)			
	73	85	90	76

Table S8. Theoretical and real mass balance (%) of ammonium ions present in the feed and receiver solutions of the experiments conducted with a real feed solution using Ralex and Fumasep membranes at different conditions (Figure 8).

Aliquot	Theoretical mass balance (%)	
	Ralex; NaCl 0.25 M; V_F:V_R of 1:2; 220 mL/min; 30 hours	Fumasep; NaCl 0.5 M; V_F:V_R of 1:2; 330 mL/min; 80 hours
1	100	100
2	98	95
3	100	89
4	93	88
5	94	90
6	95	78
7	89	80
8	92	84
9	91	84
		83
		86
		84
		88
	Real/final mass balance (%)	
	85	91

Table S9. Molar concentration (mmol/L) of trace/other cationic and anionic species present in the initial and final real feed and receiver solutions.

	Molar concentration (mmol/L)							
	Ralex; NaCl 0.25 M; V_F:V_R of 1:2; 220 mL/min; 30 hours				Fumasep; NaCl 0.5 M; V_F:V_R of 1:2; 330 mL/min; 80 hours			
	Feed (initial)	Feed (final)	Receiver (initial)	Receiver (final)	Feed (initial)	Feed (final)	Receiver (initial)	Receiver (final)
	Cationic species							
Aluminium	0.0063	0.0040			0.0059			
Boron	0.0143	0.0155			0.0132	0.0125		
Calcium	3.7034	1.2774	0.0040	0.7060	3.4126	0.5187	0.0056	1.9477
Copper	0.0026	0.0021			0.0063	0.0022		
Iron	0.0080	0.0070			0.0089	0.0079		0.0054
Magnesium	1.5509	0.9366	0.0012	0.5491	1.4399	1.0400	0.0028	0.6006
Potassium	13.276	6.3512	0.2657	2.5493	11.504	1.0391	0.4767	9.6042
Sodium	28.985	43.389	195.57	202.92	26.318	63.373	444.39	481.68
Silicon	0.1189	0.1178	0.0026	0.0025	0.1216	0.1199	0.0051	0.0061
Strontium	0.0046	0.0014			0.0047			0.0015
Zinc	0.0326	0.0195	0.0007	0.0054	0.0315	0.0119	0.0010	0.0088
	Anionic species							
Bromide	0.0261	0.0262	0.0069	0.0064	0.0272	0.0273	0.0087	0.0085
Chloride	43.206	42.426	236.42	234.38	40.836	42.368	472.37	469.20
Fluoride	25.068	22.702			21.702	21.326		
Nitrate	0.0255	0.0247	0.0199	0.0235	0.0251	0.0259	0.0201	0.0224
Nitrite	1.9828	1.9507			2.1014	2.3117		
Sulfate	0.4479	0.4405	0.0063	0.0057	0.4470	0.4603	0.0078	0.0063

Section S1. Membrane characterization by means of ATR-FTIR

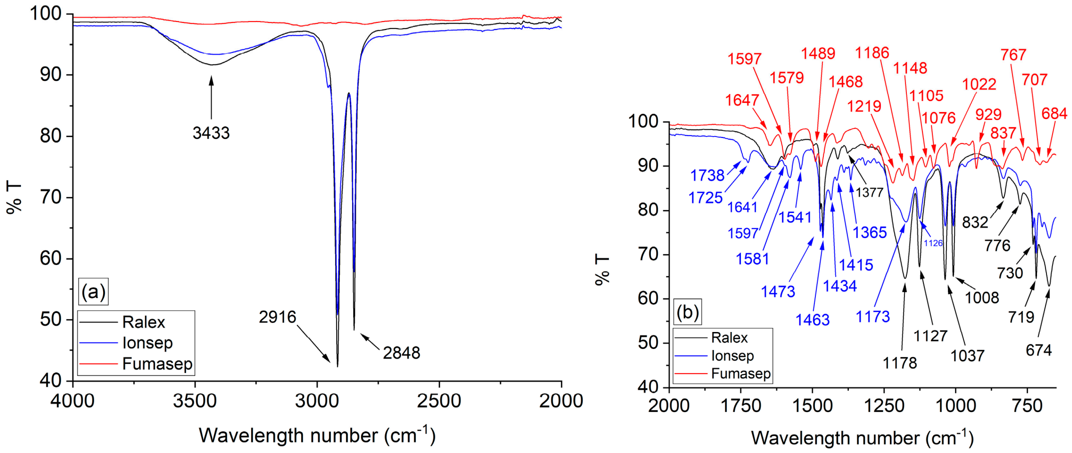

Figure S1. IR spectra of Fumasep, Ralex and Ionsep membranes.

The band at 3433 cm^{-1} present at Ionsep and Ralex is related to ν(O–H) (stretching vibration) of the bounded water [47,48]. The peaks shown for Ralex and Ionsep at 2916 cm^{-1} and 2848 cm^{-1} correspond to the $\nu_{as}CH_2$ and $\nu_{s}CH_2$, respectively [49-50]. The peaks at 1738 cm^{-1} [51] and 1725 cm^{-1} [52] correspond to the carbonyl stretching vibration ν(C=O) and are present only at Ionsep. The band at 1641 cm^{-1} present at Ralex and Ionsep is due to the stretching of CH- groups [53]. The bands at 1597 cm^{-1} for Ionsep and Ralex and 1598

cm^{-1} for Fumasep correspond to the aromatic C=C stretching in the polymer matrix [54]. The peaks at 1581 cm^{-1} (Ionsep) and 1579 cm^{-1} (Fumasep) correspond to vibration of aromatic ring skeleton [55] and is not present at Ralex. The band at 1541 cm^{-1} is present only at Ionsep and is related to the C=C vibration [54]. The peaks at 1473 cm^{-1} for Ralex and Ionsep and 1468 cm^{-1} for Fumasep also correspond to the aromatic C=C [56]. The band at 1463 cm^{-1} for Ralex and Ionsep is due to the asymmetrical CH_3 bending vibration [50,57]. The peak at 1434 cm^{-1} is present only at Ionsep and is related to the symmetric stretching vibration of $–CH_2–$ [58,59]. The peaks at 1415 cm^{-1} for Ionsep and Fumasep and 1413 cm^{-1} for Ralex correspond to the aromatic C-H stretching vibrations [60]. The peak at 1365 cm^{-1} for Ionsep and 1377 cm^{-1} for Ralex are related to the δC-H [61,62]. A peak at 1219 cm^{-1} was verified only for Fumasep and corresponds to S=O stretches belonging to sulfonic acid groups [63]. The bands at 1173 cm^{-1}, 1126 cm^{-1}, 1037 cm^{-1}, 1008 cm^{-1} for Ionsep, and 1178 cm^{-1}, 1127 cm^{-1}, 1037 cm^{-1} and 1008 cm^{-1} for Ralex correspond to the $-SO_3^-$ functional groups [64-67]. The band at 929 cm^{-1} is present only at Fumasep and is related to C-O groups [68-70]. The peaks at 837 cm^{-1} for Fumasep and 832 cm^{-1} for Ralex and Ionsep are due to C-H bending, as well as the peaks at 776 cm^{-1} (Ralex and Ionsep) and 767 cm^{-1} (Fumasep) [71]. The bands at 730 cm^{-1} and 719 cm^{-1} were verified for Ralex and Ionsep and correspond to the CH_2 deformation [67]. The peak at 707 cm^{-1} was present only at Fumasep and corresponds to C-H stretching [72]. The band at 674 cm^{-1} is related to the symmetric stretching vibration of S-O groups and is present only at Ralex and Ionsep membranes [73]. A small peak was verified for Fumasep at 684 cm^{-1}, which may also be related to the aromatic C-H out-of-plane deformation [74].

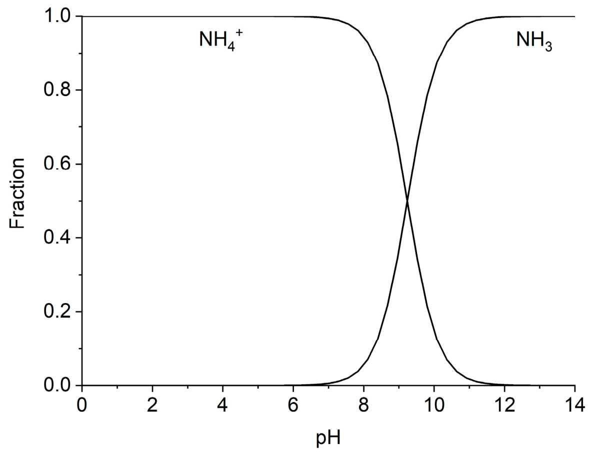

Figure S2. Speciation diagram for ammonia/ammonium ions.
